# Supplementary material for: Genome-Wide Characterization of Calmodulin and Calmodulin-like Protein Gene Families in Paulownia fortunei and Identification of Their Potential Involvement in Paulownia Witches’ Broom
Source: Genes (Basel). 2023 Jul 27;14(8):1540. doi: 10.3390/genes14081540 (PMC10454933; doi:10.3390/genes14081540)
Supplement: Supplementary file 1 [file genes-14-01540-s001.zip › genes-2413807-supplementary/Supplementary table 1.Genes of CaMCML in Arabidopsis thalina.pdf]

Supplementary table 1. CaM/CML gene from *Arabidopsis thaliana* were used for identification.

| **Gene symbol** | **Gene locus** | **Gene symbol** | **Gene locus** |
|---|---|---|---|
| AtCaM1 | AT5G37780 | AtCML23 | AT1G66400 |
| AtCaM2 | AT2G41110 | AtCML24 | AT5G37770 |
| AtCaM3 | AT3G56800 | AtCML25 | AT1G24620 |
| AtCaM4 | AT1G66410 | AtCML26 | AT1G73630 |
| AtCaM5 | AT2G27030 | AtCML27 | AT1G18210 |
| AtCaM6 | AT5G21274 | AtCML28 | AT3G03430 |
| AtCaM7 | AT3G43810 | AtCML29 | AT5G17480 |
| AtCML1 | AT3G59450 | AtCML30 | AT2G15680 |
| AtCML2 | AT4G12860 | AtCML31 | AT2G36180 |
| AtCML3 | AT3G07490 | AtCML32 | AT5G17470 |
| AtCML4 | AT3G59440 | AtCML33 | AT3G03400 |
| AtCML5 | AT2G43290 | AtCML34 | AT3G03410 |
| AtCML6 | AT4G03290 | AtCML35 | AT2G41410 |
| AtCML7 | AT1G05990 | AtCML36 | AT3G10190 |
| AtCML8 | AT4G14640 | AtCML37 | AT5G42380 |
| AtCML9 | AT3G51920 | AtCML38 | AT1G76650 |
| AtCML10 | AT2G41090 | AtCML39 | AT1G76640 |
| AtCML11 | AT3G22930 | AtCML40 | AT3G01830 |
| AtCML12 | AT2G41100 | AtCML41 | AT3G50770 |
| AtCML13 | AT1G12310 | AtCML42 | AT4G20780 |
| AtCML14 | AT1G62820 | AtCML43 | AT5G44460 |
| AtCML15 | AT1G18530 | AtCML44 | AT1G21550 |
| AtCML16 | AT3G25600 | AtCML45 | AT3G29000 |
| AtCML17 | AT1G32250 | AtCML46 | AT5G39670 |
| AtCML18 | AT3G03000 | AtCML47 | AT3G47480 |
| AtCML19 | AT4G37010 | AtCML48 | AT2G27480 |
| AtCML20 | AT3G50360 | AtCML49 | AT3G10300 |
| AtCML21 | AT4G26470 | AtCML50 | AT5G04170 |
| AtCML22 | AT3G24110 | | |
