# Supplementary material for: Genome-Wide Characterization of Calmodulin and Calmodulin-like Protein Gene Families in Paulownia fortunei and Identification of Their Potential Involvement in Paulownia Witches’ Broom
Source: Genes (Basel). 2023 Jul 27;14(8):1540. doi: 10.3390/genes14081540 (PMC10454933; doi:10.3390/genes14081540)
Supplement: Supplementary file 1 [file genes-14-01540-s001.zip › genes-2413807-supplementary/Supplementary table 3. Orthologous gene pairs within Paulownia fortunei..pdf]

Supplementary table 3. Orthologous gene pairs within *Paulownia fortunei*.

**Gene pair**	**Ka**	**Ks**	**Ka/Ks**
PfCaM1 & PfCaM3	0.002799817	0.532584536	0.005257037
PfCML3 & PfCML58	0.082598118	0.624698454	0.132220782
PfCML5 & PfCML22	0.181900751	1.58783172	0.114559212
PfCML6 & PfCML29	0.054898145	0.274407135	0.200060922
PfCML9 & PfCML30	0.101338948	0.562858584	0.180043356
PfCML11 & PfCML34	0.114189206	0.474069019	0.240870425
PfCML11 & PfCML37	0.512831281	2.478986707	0.206871331
PfCML12 & PfCML44	0.152866087	0.291575854	0.524275535
PfCML14 & PfCML43	0.0743213	0.676326031	0.109889752
PfCML15 & PfCML45	0.038112078	0.533541903	0.071432211
PfCML17 & PfCML36	0.008383879	0.325186018	0.025781793
PfCML18 & PfCML42	0.052182695	0.327821739	0.159180094
PfCML19 & PfCML35	0.090946641	0.440266056	0.206572003
PfCML20 & PfCML49	0.01624479	0.663851673	0.024470511
PfCML21 & PfCML46	0.094362603	0.557123495	0.16937466
PfCML24 & PfCML33	0.474775434	1.903278627	0.249451356
PfCML24 & PfCML40	0.246826559	0.809663993	0.304850606
PfCML28 & PfCML47	0.171866549	5.524359628	0.031110674
PfCML50 & PfCML57	0.004543747	0.016108002	0.282080137
PfCML51 & PfCML53	0.044145138	0.098935755	0.446200042
PfCML51 & PfCML55	0.08699095	0.101951154	0.85326106
PfCML52 & PfCML54	0.051690415	0.11136	0.464173989
PfCML52 & PfCML56	0.025400998	0.028466415	0.892314623

Notes: Ka, non-synonymous substitution rate; Ks, synonymous substitution rate
