# Supplementary figures and images for: Genome-Wide Characterization of Calmodulin and Calmodulin-like Protein Gene Families in Paulownia fortunei and Identification of Their Potential Involvement in Paulownia Witches’ Broom
Source: Genes (Basel). 2023 Jul 27;14(8):1540. doi: 10.3390/genes14081540 (PMC10454933; doi:10.3390/genes14081540)

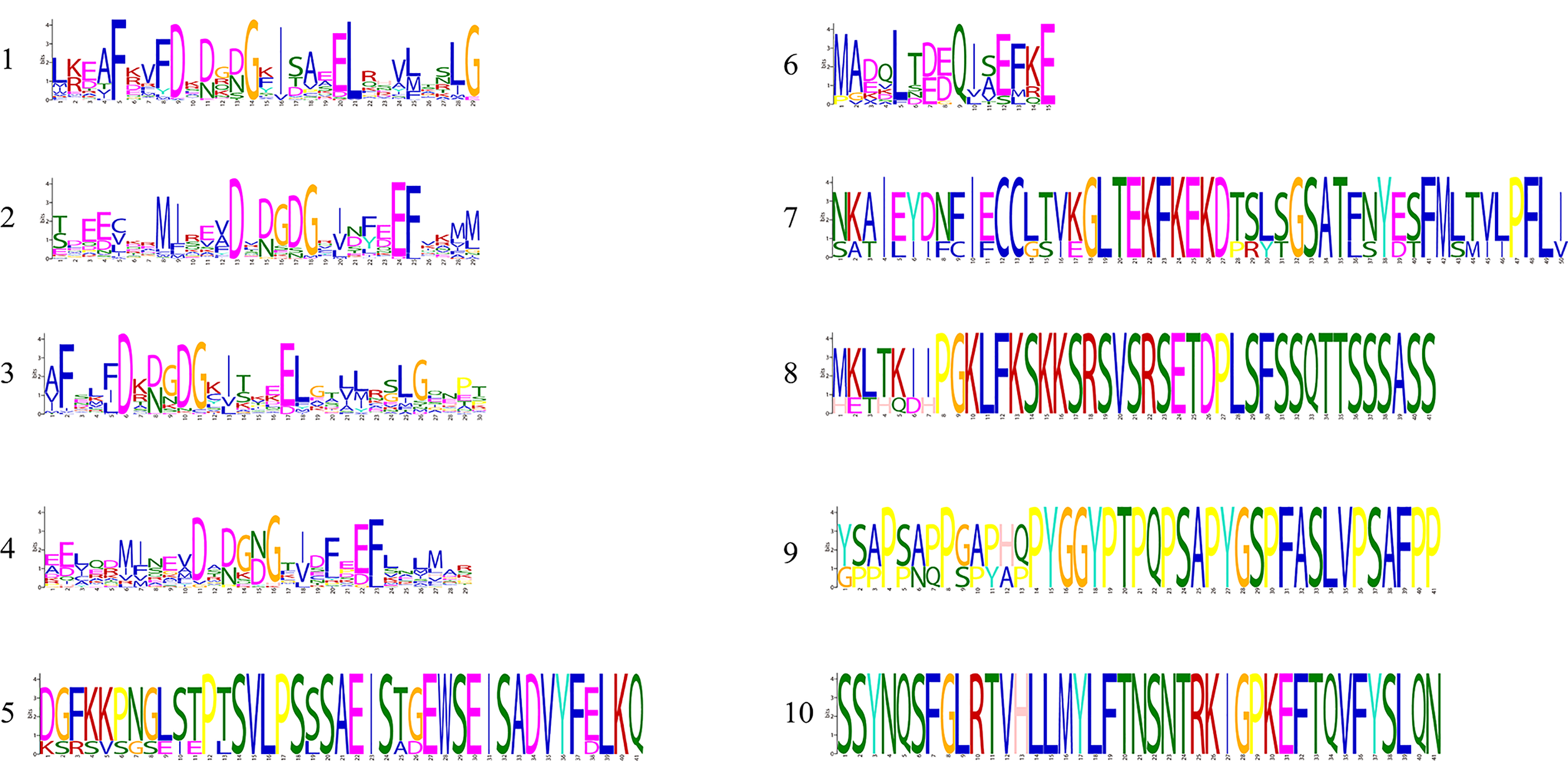

Supplement: Supplementary file 1 [file genes-14-01540-s001.zip › genes-2413807-supplementary/Supplementary Figure 1 Sequences LOGO of the PfCaMCMLs protein motif.tif]

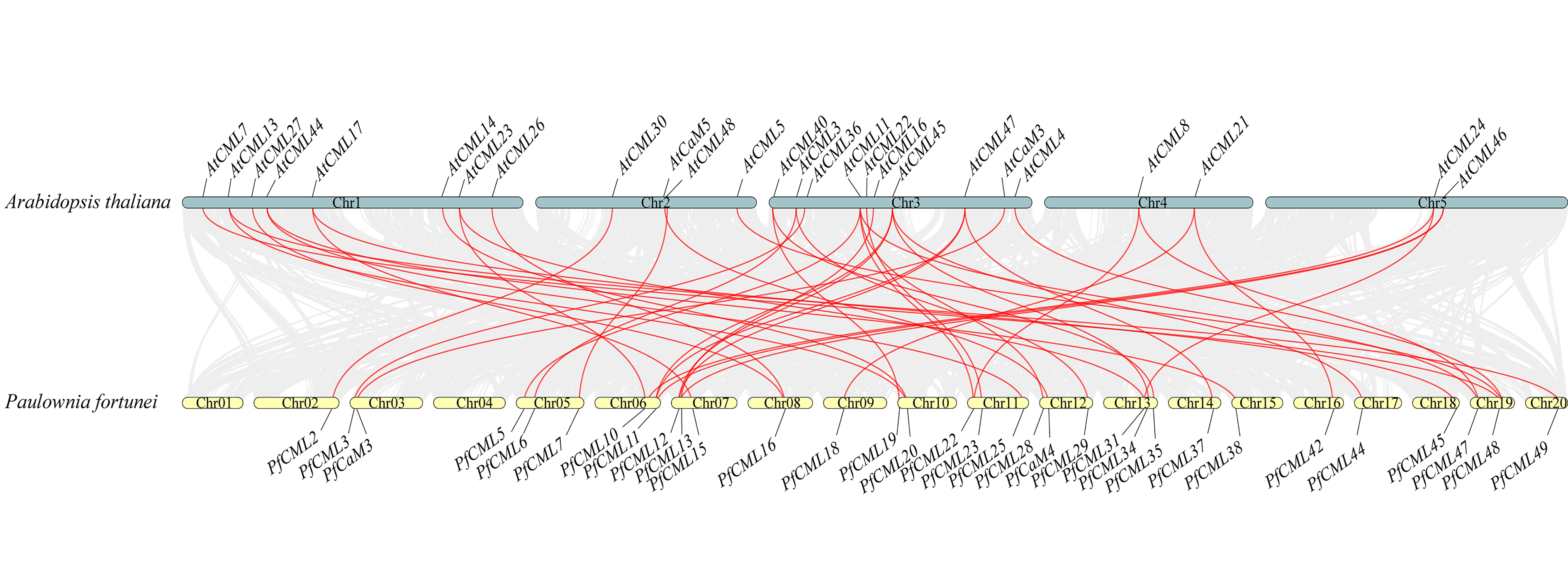

Supplement: Supplementary file 1 [file genes-14-01540-s001.zip › genes-2413807-supplementary/Supplementary Figure 2 Collinearity analysis of between Paulownia fortunei and Arabidopsis thaliana.tif]
